# Giant Primary Cutaneous Nodular Melanoma of the Forehead: A Case Report

**DOI:** 10.3390/geriatrics9060164

**Published:** 2024-12-16

**Authors:** Samantha Montandon, Charles Jefferson-Loveday, Matthew Sommerlad, Harnish P. Patel

**Affiliations:** 1 Medicine for Older People, University Hospital Southampton, Tremona Road, Southampton SO16 6YD, UK; samantha.montandon@uhs.nhs.uk (S.M.);; 2 Department of Histopathology, University Hospital Southampton, Tremona Road, Southampton SO16 6YD, UK; 3 NIHR Southampton Biomedical Research Centre, University of Southampton, Southampton SO16 6YD, UK; 4 Academic Geriatric Medicine, University of Southampton, Southampton SO16 6YD, UK

**Keywords:** giant nodular melanoma, older person, surgical resection

## Abstract

**Background:** The incidence of melanoma is increasing globally. The estimated worldwide incidence is projected to increase from 324,635 cases in 2020 to 510,000 in 2040. In the UK, melanoma accounts for 4% of all new cases of cancer. Melanomas occurring in the skin of the head and neck represent 13% and 23% of cases in women and men, respectively. Prognostic indicators include presence of nodal or distant metastasis, ulceration, and Breslow thickness, where >4 mm thickness predicts poorest overall survival rates. Giant melanomas, a term generally applied to melanomas larger than 5–10 cm, are rare and often have a very poor prognosis. **Clinical case:** An 82-year-old female presented acutely with a 2–3-day history of delirium and urinary retention in February 2022. In addition, she was noted to have a large fungating growth on her forehead that obscured the bridge of the nose and had been slowly increasing in size for the past year prior to admission. She had initially presented in primary care with a small growth on her forehead but declined further investigations for fear of contracting COVID-19. She consented to having further assessment and management of the forehead mass. A shave biopsy revealed giant nodular melanoma, specifically, the largest melanoma of the face reported in the literature. Remarkably, our patient underwent a successful complete excision and skin grafting, with no evidence of recurrence or distal metastasis after 2 years of follow up. **Conclusions:** This case highlights the anxieties people felt about contracting COVID-19 when national guidelines recommended shielding that had resulted in further morbidity. Despite poor prognostic factors, clinically and histologically, our patient did not need any systemic anticancer therapy nor radiotherapy. She was well after 2 years follow up without any signs of recurrence.

## 1. Introduction

This unique case describes a rare case of a giant nodular melanoma of the forehead in which effective shared decision-making amongst the multidisciplinary team (MDT) with our patient was key to managing the large facial tumour both in the acute setting and in the community. This case report has been written in conjunction with the CARE (CAse REport checklist) [1].

## 2. Case Presentation

**Figure 1:**
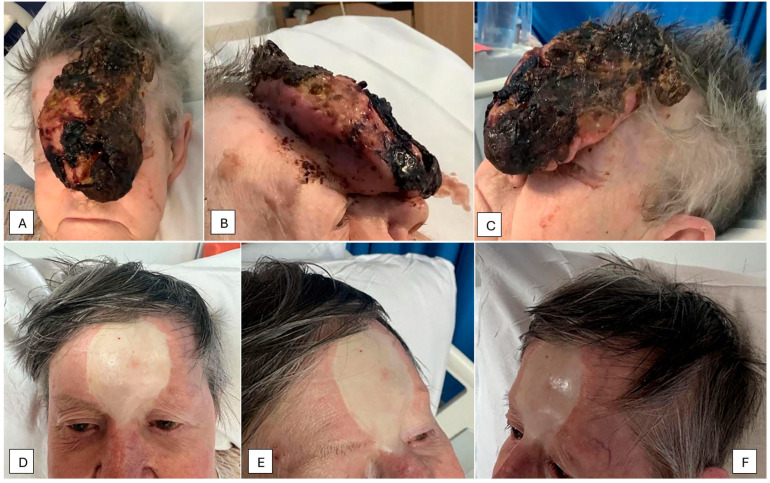
Preoperative and post operative images of the 120 × 80 × 30 mm Melanoma (**A**–**C**). Post-operative skin graft (**D**–**F**), taken 2 years after the procedure.

**Figure 2:**
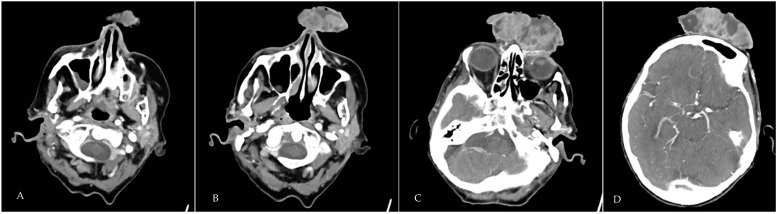
(**A**–**D**) Select Computerised Tomography slices showing the position and extent of the melanoma. There was no evidence of erosion into the frontal bone.

In February 2022, an 82-year-old independently active, Caucasian woman with fair skin was referred to the emergency department by her general practitioner (GP) with confusion and urinary retention. Her past medical history included hypertension for which she was prescribed amlodipine 5 mg once daily. She also had a large facial lesion and presented to her GP one year prior to admission with a small 1-centimetre growth on her forehead but had declined further investigation due to concerns and heightened anxiety over contracting COVID-19. She lived on her own, did not drink excess alcohol, and did not smoke. She did not have any personal of family history of melanoma or any other skin cancers. On admission, she had a large fungating mass obscuring her forehead and bridge of the nose (Figure 1A–C). Although she previously declined investigation in the community, she consented to having further assessment of the growth as an inpatient alongside active management of the delirium and urinary retention in a shared decision-making process. Her admission through discharge blood serum values are shown in Table 1. Our patient was anaemic with blood values that confirmed iron and vitamin B12 deficiency. In addition, she was also depleted in Vitamin D. Imaging and point of care testing did not reveal any focus of infection in her lungs or evidence of COVID-19 disease, respectively. A skin swab around the facial growth revealed *Staphylococcus aureus* that was sensitive to flucloxacillin. Subsequently, one to two weeks after her admission, she contracted COVID-19 and was treated with Remdesivir. She developed atrial fibrillation that was managed appropriately with Digoxin, beta-adrenoreceptor blockade, and a direct oral anticoagulant (DOAC). She also received ferric derisomaltose, a 2-unit blood transfusion as well as Vitamin D and B12 replacement. An urgent shave biopsy performed by the maxillofacial team 8 days after admission confirmed a diagnosis of melanoma. A CT scan of the head, neck, and chest with contrast showed no evidence of erosion of the tumour into the frontal bone or distal metastasis (Figure 2A–D). However, it did show an incidental finding of bilateral pulmonary emboli that was managed with the existing DOAC prescription. Following shared decision-making discussions between the multidisciplinary team (MDT) and our patient, a palliative excision of the melanoma was performed in March 2022.

**Figure 3:**
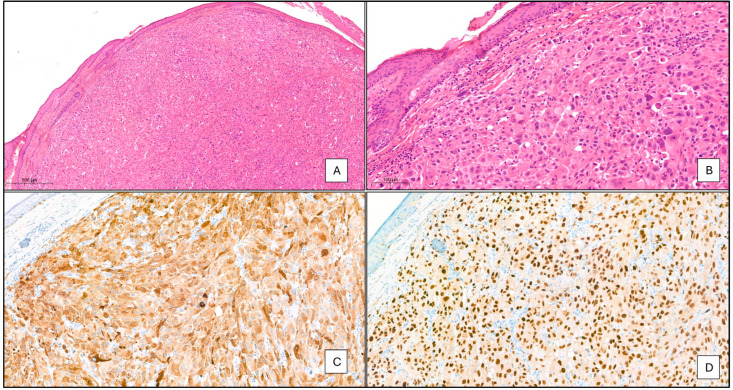
(**A**) (×5 magnification) and (**B**) (×20). These show part of a tumour consisting of atypical epithelioid cells with pleomorphic nuclei, prominent nucleoli, and occasional intranuclear inclusions. Immunohistochemistry revealed positive expression of the melanocytic markers Melan A (**C**) (×20), SOX10 (**D**) (×20).

The melanoma was excised down to the pericranium with a 2 mm radial margin and 0.1 mm deep margin. A full thickness skin graft from her right iliac fossa was applied to her forehead. Subsequent histological examination of the tumour revealed a 120 × 80 × 30 mm exophytic tumour confirmed as an ulcerated nodular melanoma with a Breslow thickness of at least 30 mm, vertical invasion and pathological staging of pT4b N0 M0. Mitotic count was 4/mm^2^. Importantly, there was no lymphovascular or perineural invasion. Histology revealed atypical epithelioid cells with pleomorphic nuclei, prominent nucleoli, and occasional intranuclear inclusions (Figure 3A,B). The tumour did not arise from an underlying dysplastic naevus. Immunohistochemistry showed positive expression of melanocytic markers (Melan A, SOX10 (Figure 3C,D) as well as S100 and HMB45) but negative for CD 10, MNF 116, CK5/6, P63, SMA, Desmin, and Caldesmon. There were focal in situ components consisting of single atypical melanocytic cells along the dermoepidermal junction. Mutation analysis revealed the presence of wild type *BRAF* gene whilst BRAF Val600Glu (BRAF V600E) and Val600Lys (BRAF V600K) activation variants were not detected.

**Figure 4:**
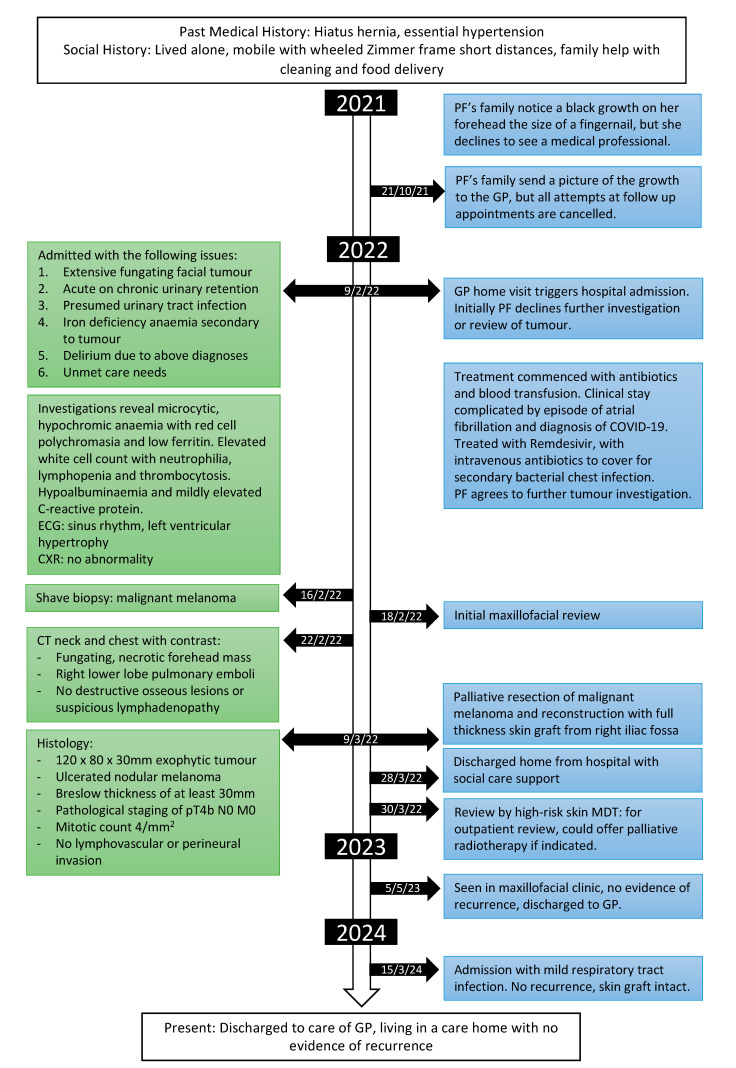
Timeline showing key points in our patient’s journey from admission, discharge, and follow up.

Our patient was reviewed by the high-risk skin multidisciplinary team (MDT) 30/3/22 and the case was overseen by the maxillofacial team who arranged follow up and considered offering palliative radiotherapy. However, one year post excision, she was asymptomatic and therefore no further treatment or investigation was recommended. She was subsequently discharged to the care of her GP with the following medications: Apixaban 5 mg twice a day, Bisoprolol 2.5 mg once a day, Amlodipine 5 mg once a day, and Lansoprazole 30 mg once a day. She was admitted for a minor respiratory tract infection in March 2024, two years after the excision. She had no evidence of tumour recurrence, and the skin graft was intact (Figure 1D–F). The timeline illustrating our patient’s admission to follow up is shown in Figure 4.

## 3. Review

This case describes an older independent woman diagnosed with a large, ulcerating, nodular melanoma and underwent a successful excision that needed application of a full-thickness skin graft. Our patient had several poor prognostic indicators: older age; head and neck situation of the tumour; presence of tumour ulceration; and a Breslow thickness of at least 30 mm. However, fortunately for our patient, there was no evidence of local or distant metastasis or recurrence 2 years later.

Melanoma occurs due to the malignant transformation of melanocytes commonly found in the epidermis of the skin, hair follicles, and the uvea but also many other sites due to their origination from the cells of the neural crest [2]. Melanoma in the skin usually begins with a radial growth phase, where the malignant cells spread across the epidermis (melanoma in situ). At this stage excision of the lesion is curative and prognosis is favourable. Eventually, there is progression to the vertical growth phase, where malignant cells invade the dermis conferring their metastatic potential [2]. The underlying mechanisms of melanomagenesis are multifactorial and complex, and a detailed summary can be found in reviews by Demierre et al. [3] and Guo et al. [4]. However, it is worth briefly mentioning some important driver mutations, including mutations of the *BRAF* oncogene, that are present in 60% of all cutaneous melanomas, mutation in the oncogene NRAS and the tumour suppressor gene neurofibromatosis 1 (NF1). These driver mutations ultimately result in hyperactivation of intracellular signal transduction such as extracellular signal regulated kinases (ERK) that lead to abnormal cell proliferation and differentiation. For example, the mitogen-activated protein kinase (MAPK) signalling pathway appears to be a main factor in melanomagenesis as up to 90% of melanomas display MAPK pathway hyperactivation. The most common BRAF mutation causes a valine to glutamic acid substitution at position 600 producing the mutated protein BRAF V600E. However, mutations in *BRAF* alone are not enough to produce melanoma. Indeed, BRAF mutations can be found in up to 80% of benign nevi where the melanocytes have proliferated but remain senescent [5]. Ultraviolet (UVA and UVB) light radiation from sunlight exposure to the skin is a significant contributor to melanocyte DNA damage and melanomas in skin exposed areas tend to exhibit a higher mutation load as a consequence of dysregulation of DNA repair within melanocytes [6].

The classification of melanoma is based on histological subtypes and includes superficial spreading melanoma; nodular melanoma; lentigo maligna melanoma; and acral lentiginous melanoma. After superficial spreading melanoma, nodular melanoma is the second most common subtype [2] and confers a poor prognosis secondary to rapid growth and the fact that it appears to have only a vertical growth phase, meaning Breslow thickness is likely to be increased at the point of diagnosis [3]. Risk factors for melanoma include a personal or family history of melanoma, excessive sun exposure (ultraviolet radiation), previous severe sunburn as a child or teenager, presence of a cancer-prone syndrome (such as xeroderma pigmentosum or familial atypical mole syndrome), and immunosuppression. In addition, geographical area, atmosphere, latitude, and cloud cover influence the exposure to UV radiation. The patient’s phenotype is also important. Those with lighter skin or eye colour, red or blond hair, and the presence of a large amount of nevi are at an increased risk [7]. A moderate degree of heritability is also suggested e.g., BRCA-1 mutation. It should be noted that the majority of melanomas arise de novo rather than from an existing nevus [8]. Our patient was Caucasian, with light skin and brown iris colour. She was unable to recall severe sunburn when she was younger but denied any personal or family history of skin cancer.

Definitive diagnosis of melanoma is primarily histopathological, and excision biopsies are preferred as this allows measurement of the thickness of the tumour which informs staging and further management. Breslow thickness is an important prognostic indicator where increasing thickness correlates to lower 5-year overall survival [9,10]. Our patient had a T4b N0 M0 tumour which translates to stage IIC disease with an overall predicted 5-year survival rate of 82%. Other poor prognostic factors are advancing age; male sex; anatomical location such as the head and neck or trunk; nodular melanoma; and presence of lymphatic invasion within the tumour [11]. Treatment involves excision of the tumour, where possible, with a clinical margin of 0.5–2 cm depending on staging [12]. Localised disease can be treated with surgical resection, but this is not curative for advanced disease. Melanoma management is challenging as this is a solid tumour with a high number of mutations conferring potential for tissue invasion and metastatic spread. Progression of melanoma is invariably linked to the lack of activation and exhaustion of the immune system with down regulation of tumour associated antigens such as glycoprotein 100, tyrosine and Melan-A. However, over the last 10–15 years, detailed knowledge and understanding of the pathogenesis of melanoma has informed the development of targeted therapies that alone, in combination or in sequence, have revolutionised the management of melanoma. In terms of guidance on when, who and how to treat, the UK National Institute for Health and Care Excellence (NICE) guidelines recommend treatment according to disease stage are summarised succinctly in Table 2. Fortunately, there was no indication for systemic anticancer therapy in our patient.

## 4. Discussion

To the best of our knowledge, this is the largest case of facial melanoma recorded at 120 × 80 × 30 mm, and one of the minority of cases without nodal or metastatic spread. In 2014, Di Meo et al. [13] published a comprehensive review of the literature which found 16 cases of giant melanoma (>10 cm). Only one case described a melanoma of the skin of the face: it was located on the eyelid measuring 50 × 45 × 40 mm. Comparing to other head and neck melanomas there were three cases described which involved the scalp, one of which total measurement was not given, the other two measured 120 × 100 mm and 145 × 104 mm [13,14,15]. In 2020, Megna et al. published a case of a 52 × 20 mm facial melanoma of the nose that had been slowly growing for 15 years. The patient refused complete surgical resection, radiotherapy, and sentinel lymph node biopsy and was lost to follow up at 5 months [16].

From our review of the literature, it is extremely rare for a patient with a giant melanoma to present without any metastatic spread or even lymphovascular invasion. In the review of the literature by Di Meo et al. [13], all but one case described people with stage III or VI disease; Panajotovic et al. [15] published a case of a giant scalp melanoma weighing 453 g without nodal or distant metastatic spread in 2007. Unfortunately, three weeks after tumour resection and skin grafting, the patient died of a myocardial infarction. Two further UK cases of giant melanoma at stage IIC without nodal or distant metastases were published by Honeyman et al. and Faderani et al. [17,18]. Delayed presentation was a common feature on review of the literature [19]. Faderani et al. [18] published a case of a patient in the UK with a 77 × 77 × 54 mm thigh giant malignant melanoma, whose presentation was also delayed due to fear of hospital settings in the COVID-19 pandemic. It is unsurprising that psychological factors play an important role in the deferral of seeking medical attention, as described by Honeyman et al. [17] who presented a further case of a 14 × 7 × 12 cm upper limb melanoma in a patient in the UK who avoided hospitals due to severe anxiety. It is therefore essential for clinicians to be aware of these patient factors as it could also influence concordance and suitability for further treatment and follow up. Such is the rarity and heterogeneity of giant melanoma, there is no specific deviation from usual practice.

The exact reason why our patient did not have local or distant metastases on assessment and follow up is not completely understood. However, there may be selective patient, tumour location and molecular determinants, including genetic and epigenetic alterations that remain unexplored. We know our patient lived alone and was supported by her daughter. Her nutritional intake was not assessed in the community but improved when was in a supportive environment in hospital and was aided by the provision of oral supplements. The tumour site abutting the pericranium (Figure 3) is unlikely to have contributed to the selective external growth as skin coverings of the pericranium are rich in vascular structures and connective tissue and local invasion would have been expected from the size, location and histology. However, we do know that there was no direct pericranial invasion (Figure 3). Furthermore, she was negative for the antigens/cytoskeletal proteins CD 10, MNF 116, CK5/6, P63, SMA, Desmin, and Caldesmon, that, in unison with absence of MAPK or BRAF mutations, may have conferred lower metastatic potential [20,21,22,23,24]. From the molecular point of view, metabolic rewiring of melanoma is a well described phenomenon that allows melanoma cells to adapt to hypoxaemia and nutrient deprivation through alterations in the glycolysis, tricarboxylic, and oxidative phosphorylation pathways, invariably involving metabolic and morphological changes to mitochondria [25,26]. Here, melanoma cells are better suited to managing oxidative stress and metabolising lactate by elevating the transporter protein MCT-1 [27]. In this regard, higher levels of oxidative stress may be associated with lower metastatic potential. It is tempting to speculate that our patient did have higher levels of metabolic stress as evidence by the friable nature of the tumour and the extensive areas of necrosis as seen in (Figure 1A–C and Figure 2). Further detailed discussion about the factors predicting metastases and molecular work on putative factors determining metastatic spread is out of scope of this case report. However, readers are directed to a few comprehensive publications on this topic [25,27,28].

## 5. Conclusion and Learning Points

Giant melanoma predominantly refers to melanoma larger than 5–10 cm in size. Patients with giant melanoma often present late and it is important to be cognisant to underlying psychological factors that have influenced this such as was the case in our patient.

Hospital admission of our patient by her GP represented a turning point for her. Within a few days of admission, and following treatment for her immediate medical illnesses, our patient was able to overcome some of the psychological barriers that previously prevented her from consenting to further investigation and management of the growth and developed trust in her treating teams. This was dependent on non-judgmental compassionate care, regular support from all members of the multidisciplinary team, and attention to her individual needs, as assessed by comprehensive geriatric assessment (GCA) [29]. These factors were undoubtedly key to a successful therapeutic relationship. A complete and honest explanation of treatment options for the melanoma were given, facilitating a shared decision-making process between the multidisciplinary team, the patient and her appointed contacts/next of kin to arrive at a comprehensive plan for treatment, discharge and follow up [30], using key principles in guidance produced by NICE (Table 2 and Table 3).

## 6. Key Points

1. Melanoma is the main cause of death from skin cancer.
2. Management of melanoma in older people should involve the MDT with the surgical, dermatology, and oncology teams to address the patient’s social and psychosocial factors, which will permit a comprehensive plan for treatment and follow up.
3. Sentinel node biopsy is recommended for melanoma with Breslow thickness of 0.8–1.0 mm with at least one of the features: ulceration, lymphovascular invasion, a mitotic index of 2 or more.
4. Tumour stage at diagnosis is the main predictor of survival approximated at 98.3% at 5 years for localised melanoma and 16% for metastatic disease.
5. Breslow thickness, lymph node involvement and presence of metastases form the basis for melanoma staging. Stages I & II refer to local disease, III & IV refer to local lymph node involvement and distant metastases.
6. There are clear published guidelines on treatment and follow up of melanoma that should be referenced during Shared Decision Making.

## Figures and Tables

**Table 1:** Serum values on admission to discharge.

	Units of Measurement	Normal-Range	Patient Values on Admission 9 February 2022	Patient Values on Discharge 24 March 2022
Haemoglobin	g/L	130–170	87	104
White cell count	10^9^/L	4.0–11.0	15.3	12.3
Platelets	10^9^/L	150–400	650	622
MCV	FL	80–100	77.6	85.2
CRP	mg/L	0–7.5	42.0	6.0
Urea	mmol/L	2.5–7.8	26.1	5.6
Creatinine	μmol/L	80–115	158	73
eGFR	mL/min/1.73 m^2^	40–320	26	66
Albumin	g/L	35–50	34	28
Lactate	mmol/L	0.5–2.0	1.6	1.4
Lactate Dehydrogenase	U/L	225–425	719	-
Vitamin D	nmol/L	<25: consistent with deficiency	<5	-
Procalcitonin	-	<0.05	0.05	-
Point of care testing			COVID-19 negative, urine analysis and culture negative	COVID-19 negative
Blood film	Hypersegmented neutrophils, microcytic hypochromic cells, polychromasia			
Iron studies	Iron < 2 (5–30), Transferrin 1.35 g/L (1.9–2.8), Ferritin 65 ug/L (11–307)			
B12/Folate	133 ng/L (>160)/4.2 ng/mL (3.8–25)			
Chest radiograph	No focal consolidation on admitting radiograph			

**Table 2:** Adapted summary of NICE Guidelines 2022 [12]—Principles of Treatment.

Stage 0 to II—Excision * with clinical margin 0.5–2 cm ≥0.5 cm—Stage 0 ≥1 cm—Stage I or where 2 cm margin unacceptable ≥2 cm—Stage II **Stage III (lymph nodes or in transit spread)** Micrometastatic nodal disease detected by SLNB—Completion lymph node dissection not routinely offered, requires discussion with patient and SS MDT Palpable stage IIIB to IIID melanoma, or cytologically/histologically confirmed nodal disease detected by imaging—therapeutic lymph node dissection offered. Stage IIIB–IIID—adjuvant radiotherapy only offered if reduction in risk of recurrence outweighs risk of adverse effects Topical imiquimod can be used to palliate superficial melanoma skin metastasis **In-transit Metastasis in stage III and IV** In transit metastasis—discuss with SS MDT Surgery is first line option If surgery not feasible or recurrence options include systemic anticancer therapy, talimogene laherparepvec *, isolated limb infusion/perfusion, radiotherapy, imiquimod **Stage IV (distant metastasis) and unresectable stage III** Oligometastatic stage IV—refer to SS MDT, consider surgery or other ablative treatments Brain metastasis—refer to SS MDT, refer to neuro-oncology MDT if possibly suitable for surgery/stereotactic radiotherapy **Systemic anticancer treatments:** Shared decision-making, encompassing full risk assessment with the person Consider co-morbidities and performance status, risks of treatment toxicity tolerance, presence of symptomatic brain metastasis, tumour biology Immunotherapy: 1st nivolumab plus ipilimumab, 2nd pembrolizumab, or nivolumab monotherapy BRAF V600 mutation-positive melanoma: if above immunotherapies are contraindicated or if not enough time for adequate immune response offer encorafenib plus binimetinib or dabrafenib plus trametinib ○ 2nd line dabrafenib or vemurafenib If targeted treatment contraindicated, consider chemotherapy with dacarbazine or best supportive care

* Imiquimod for stage 0 if excision would cause unacceptable disfigurement with repeat biopsy.

**Table 3:** Adapted summary of NICE Guidelines 2022 [12]—follow up.

**Principles** People who have completed treatment for melanoma must have been information on how to contact the SS MDT. Psychological support should be offered to the person and their families/carers at all follow up appointments. Reinforce advice about self-examination and health promotion (sun awareness, vitamin D supplementation if deficient, smoking cessation). **Routine follow up** Should involve a full examination of the skin and regional lymph nodes by trained healthcare professional with access to dermoscopy and medical photography. Alternate between CE-CT and ultrasound scans if indicated, do not routinely use PET-CT. Offer MRI instead of CE-CT to children and young adults ≤ 24 years, pregnant people, people who have known or resected brain metastasis. Offer follow-up for 1 year to people who have had stage IA melanoma, and for 5 years to people who have had stages IB to IV melanoma.

CE-CT: Contrast enhanced computed tomography, SS MDT: specialist skin cancer multidisciplinary team.

## Data Availability

Further information on this case is available by contacting the corresponding author.

## Abbreviations

MDT: Multidisciplinary team, CARE: Case Report Checklist, GP: General Practitioner, CT: Computerised Tomography, COVID-19: Severe acute respiratory syndrome coronavirus 2 (SARS-CoV-2), MAPK: Mitogen activated protein kinase, SLNB: Sentinel lymph node biopsy.
